# Supplementary material for: Fluid-derived lattices for unbiased modeling of bacterial colony growth
Source: PLoS One. 2025 Aug 28;20(8):e0330491. doi: 10.1371/journal.pone.0330491 (PMC12393729; doi:10.1371/journal.pone.0330491)
Supplement: S1 Text — (PDF) [file pone.0330491.s001.pdf]

Differences in colony morphology in simulations with iDynoMiCS and the hybrid lattice-based method

As noted in the main text, we found that iDynoMiCS [1] and the hybrid lattice-based model give substantially different colony morphologies in nutrient constrained environments. Our hybrid lattice-based model gives a branched morphology where iDynoMiCS gives a fingered morphology in nutrient poor environments, as can be seen in the bottom row of Fig 1. The iDynoMiCS model includes several features that our model does not. For example, iDynoMiCS models pressure-driven movement of bacteria and shoving between bacteria. Furthermore, the model system of iDynoMiCS is slightly different. Where we model a system akin to growth on an agar plate with a finite amount of nutrients, iDynoMiCS models a flow-cell system with a user-definable boundary layer and a nutrient concentration field fed by a bulk nutrient reservoir outside the boundary layer [1].

We compared iDynoMiCS to earlier work by Nadell *et al.* [3] to get a better understanding of the most important differences between the various models. These authors model a similar flow-cell setup with a user defined boundary layer and a bulk solute reservoir and shoving interactions between agents. Despite these similarities to the system modeled by iDynoMiCS, Nadell *et al.* do find branching colonies at low nutrient availability as opposed to the fingering morphology found by iDynoMiCS [3, 4]. The difference that stood out to us was the lack of pressure driven bacterial movement that is present in the work of Ref [3, 4]. However, disabling this pressure-driven motion in iDynoMiCS results in a fingered morphology similar to the one that we observed earlier in Fig 1. It is not clear to us which of the differences in the models causes the observed difference in colony morphology.

References

[1] Lardon LA, Merkey BV, Martins S, Dötsch A, Picioreanu C, Kreft JU, et al. iDynoMiCS: next-generation individual-based modelling of biofilms. Environmental Microbiology. 2011;13(9):2416–2434. doi:10.1111/j.1462-2920.2011.02414.x.

[2] Young E, Allen RJ. Lineage dynamics in growing biofilms: Spatial patterns of standing vs. de novo diversity. Frontiers in Microbiology. 2022;13.

[3] Nadell CD, Foster KR, Xavier JB. Emergence of Spatial Structure in Cell Groups and the Evolution of Cooperation. PLOS Computational Biology. 2010;6(3):e1000716. doi:10.1371/journal.pcbi.1000716.

[4] Bonachela JA, Nadell CD, Xavier JB, Levin SA. Universality in Bacterial Colonies. Journal of Statistical Physics. 2011;144(2):303–315. doi:10.1007/s10955-011-0179-x.

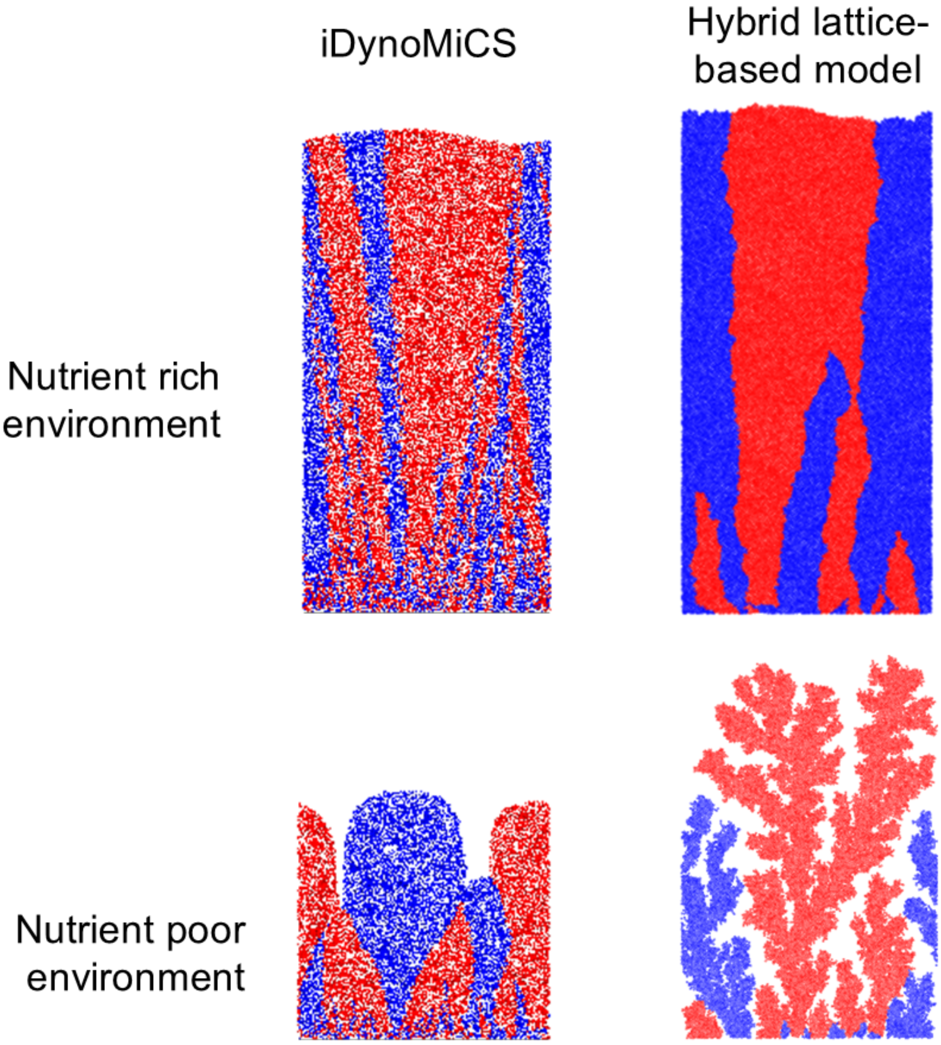

Figure 1: **The hybrid lattice-based model and `iDynoMiCS` result in different colony morphologies in nutrient poor environments.** Colonies grown under nutrient rich and nutrient poor environments using `iDynoMiCS` [1] and our hybrid lattice-based model are compared. Each simulation is seeded with 200 bacteria at the bottom of the simulation domain. Each bacterium is marked either red or blue with equal probability. Bacteria transmit this color to their daughter cells. For the `iDynoMiCS` simulations, the nutrient rich environment is set by the bulk limiting nutrient concentration $S_{\mathrm{bulk}} = 10^{-2}$ g/L and the nutrient poor environment by $S_{\mathrm{bulk}} = 10^{-3}$ g/L. The remaining parameter choices are as in Ref [2]. For the hybrid lattice-based simulations, the nutrient rich environment is set by initial nutrient concentration $c_0 = 3$ and the nutrient poor environment by setting $c_0 = 0.7$. The remaining parameter choices are as in Table 1 in the main text.
