# Supplementary material for: Fluid-derived lattices for unbiased modeling of bacterial colony growth
Source: PLoS One. 2025 Aug 28;20(8):e0330491. doi: 10.1371/journal.pone.0330491 (PMC12393729; doi:10.1371/journal.pone.0330491)
Supplement: S2 Fig — (PDF) [file pone.0330491.s002.pdf]

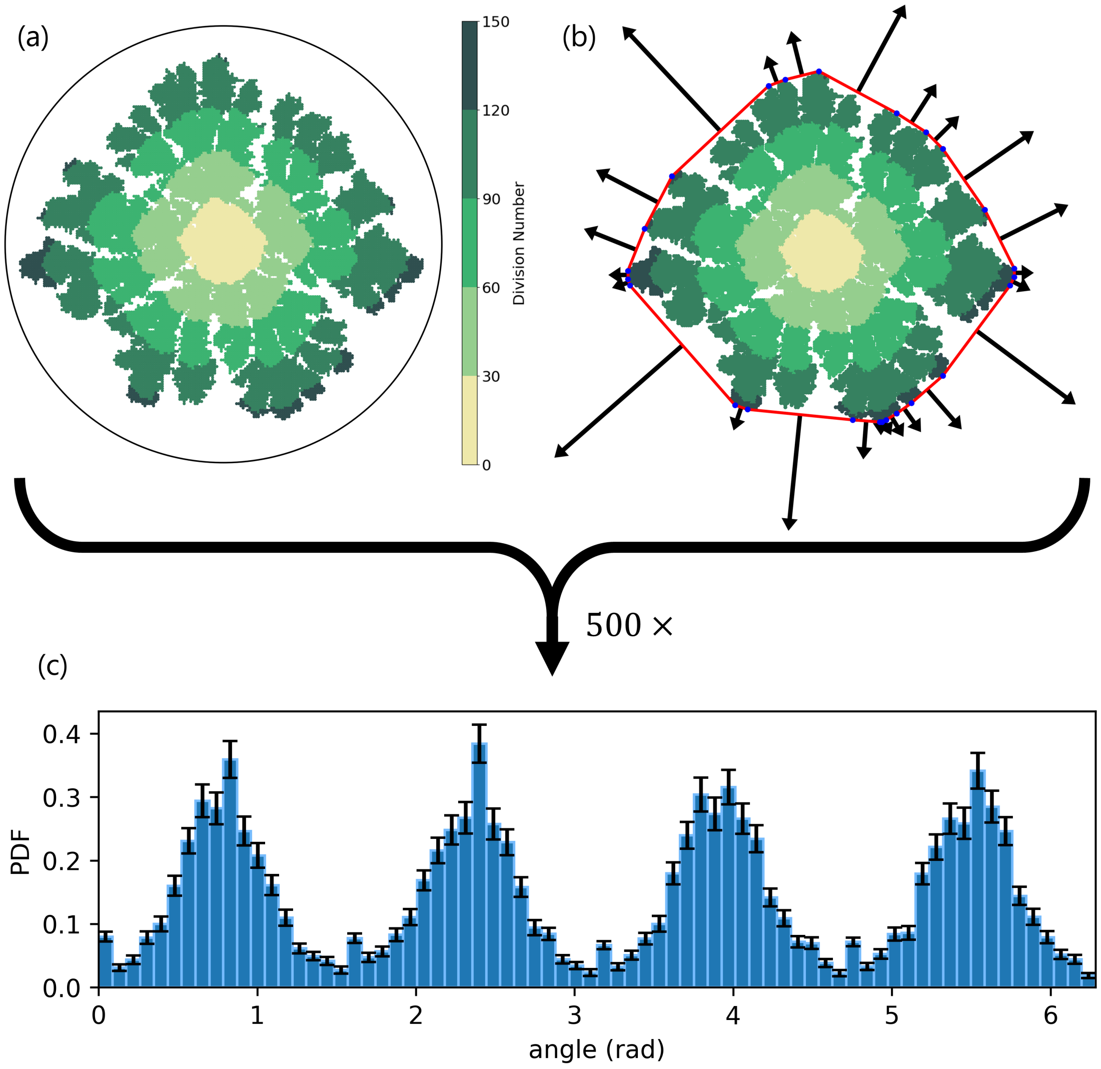

Figure 1: **Fitting of convex hulls to simulated colonies allows for quantification of lattice-induced symmetries.** (a) We perform simulations of the hybrid lattice-based model on a square lattice as an example. (b) We fit a convex hull to the final colony shape (red line). The blue dots indicate the endpoints of the convex hull segments. The normal vectors to each convex hull segment are then determined (black arrows). The weight of a normal vector (indicated by the length of the arrows) is determined by the length of its associated segment of the convex hull. This procedure is repeated for 500 independent simulations. (c) The directions and weights of all the resulting normal vectors are then collected in a histogram and normalized. Wrapping this histogram around a representative colony shape gives the figures in the main text.
